# Eustachian valve endocarditis in patients with Fournier’s gangrene and septic shock: A rare case and a literature review

**DOI:** 10.5339/qmj.2025.93

**Published:** 2025-09-15

**Authors:** Ahmed Sifeeldein Ahmed Idris, Umme Nashrah, Umm E. Amara, Nissar Shaikh

**Affiliations:** 1 Hamad General Hospital, Doha, Qatar; 2 Deccan College of Medical Sciences/Hyderabad, India; 3 Surgical Intensive Care/Hamad Medical Corporation, Doha, Qatar *Email: dr.ahmedseif1997@gmail.com

**Keywords:** Central venous catheter, coronary sinuses, Candida fungemia, *E. coli*, Fournier’s gangrene, meropenem, anidulafungin, eustachian valve, right heart endocarditis, septic shock

## Abstract

**Introduction:**

The eustachian valve (EV) is a remnant of the right sinus venosus valve. It remains different in size and shape without much impact on adult life. In 5% to 10% of all endocarditis, are seen in the right side of the heart is involved, which is rare compared to the left side of the heart. Bacteremia, central venous catheter, heart implants, and drug abuse increase the risk of EV vegetation and right heart endocarditis. We are reporting a case of EV endocarditis in patients with Fournier’s gangrene and septic shock.

**Case Presentation:**

A 45-year-old male patient was admitted into the surgical intensive care unit with Fournier’s gangrene, septic shock, and acute kidney injury (AKI). The patient was managed by invasive ventilation, noradrenaline, vasopressin, and renal replacement therapy. He developed *Escherichia coli* bacteremia and candidemia. We added meropenem and antifungal to the therapy. The transthoracic echocardiography showed EV vegetation and thread-like vegetation in the right coronary sinus, which was confirmed with transesophageal echocardiography. With aggressive therapies, the patient recovered from septic shock, organ dysfunction and was successfully liberated from invasive ventilation. The patient was discharged home on day 27. The antibiotics and antifungal were continued for 6 weeks. Two weeks after discharge, the follow-up echocardiogram was normal, and he was doing well.

**Discussion:**

Eustachian valve endocarditis is rare, and should be treated with appropriate, culture- and sensitivity-guided antibiotics and or antifungal therapy for 6 weeks. The outcome of EV vegetations of endocarditis is good. The reported mortality is up to 17%. The independent risk factors associated with mortality are AKI, the Charlson comorbidity index, congestive heart failure, larger vegetation, and central nervous system involvement.

**Conclusion:**

The presence of larger EV, along with *E. coli* (ESBL) bacteremia and fungemia, increases the risk of right-sided endocarditis, which is rarely reported. Our patient was diagnosed early, received appropriate antimicrobial treatment for a sufficient duration, resulting in a better outcome. A high index of suspicion, along with early diagnosis and culture-guided 6-week antimicrobial therapy, will improve the patient’s outcomes.

## 1. INTRODUCTION

The eustachian valve (EV) is also called “the valves of inferior vena cava” and is situated between the right atrium and the inferior vena cava opening. The EV was first described by Eustachi in the 16th century.^1^ In prenatal age, the EV helps to direct the oxygenated blood from the right atrium to the left atrium and does not have any function at the post-gestational age, and is an embryonic remnant in various sizes and minor shapes in adult life. The large EV (length >10 mm) increases the risk for thrombosis and infective vegetations, and difficulties in cardiac catheterization. Eustachian (right-sided) valve endocarditis (EVE) is comparatively lower than the left-sided endocarditis and generally reported in the form of case reports.^2,3^ Right‐sided valvular endocarditis mainly occurs in the tricuspid valve, with a described incidence of around 5% to 10% of all 48 cases of infective endocarditis.^2–4^ EVE is a rare and rarely reported clinical entity.^3,4^ *Staphylococcus aureus* accounts for the majority of cases of EV vegetation and endocarditis in patients with drug abuse and addiction.^5^ We present a case of extended-spectrum beta-lactamase-producing *Escherichia coli* (ESBL *E. coli*) and *Candida dubliniensis* endocarditis and vegetations on the EV and coronary sinuses in a patient with Fournier’s gangrene and septic shock.

The Hamad Medical Corporation Medical Research Committee has permitted the publication of this case report, and informed consent was obtained from the patient to use their data and clinical images to produce this case report (MRC number: MRC-04-24-525).

## 2. CASE PRESENTATION

**Figure 1.**
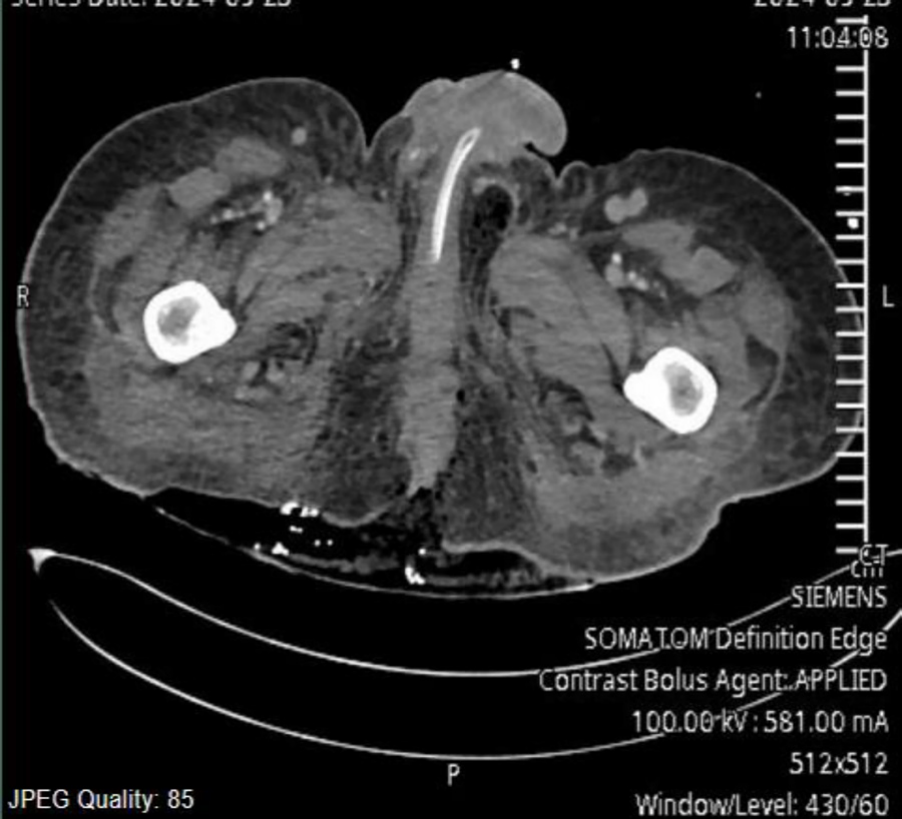
Computerized tomography scan of the pelvis showing infection along with air bubbles.

A 45-year-old Sudanese gentleman was brought to the emergency department of our hospital by ambulance with a history of diarrhea and vomiting for 3 days. His history was significant for transverse myelitis (paraplegic; he was not on immunosuppressive medication), and he was wheelchair bound. He was hypotensive (70/32 mm Hg), tachycardic (112/min), and tachypneic (28/min). Immediately started bolus of crystalloids (1,500 mL), followed by vasopressors, noradrenaline infusion. He was looking sick. His scrotum was swollen, skin was macerated, and the penis was edematous. There was a sacral bed sore with necrotic tissue. Resuscitation was continued, inserted an invasive line (right internal jugular central line and arterial line) was inserted. He had leukocytosis (26.6 × 10^9^/L), he was coagulopathic (INR 2.2, PT 25.5, APTT 36.1 seconds), renal function was impaired (BUN 13.3 mg/dL, creatinine 28.7 µmol/L), and he was hyponatremic (129 mEq/L). The sepsis markers were raised (CRP 301 mg/dL, PCT 6.9 ng/mL, and lactate 2.9 mmol/L), continued resuscitation his LRINEC score (laboratory risk indicator for necrotizing fasciitis) was 10 (CRP: 4 points, leukocytosis = 2, hyponatremia = 2 and raised serum creatinine = 2) suspected Fournier’s gangrene and planned immediate debridement, meanwhile computer tomography pelvis showed features of Fournier’s gangrene with small air foci seen perinium with edema (Figure 1). He was started on Tazocin^®^ 4.5 g every 6 hours and clindamycin, 600 mg 8 hourly intravenously. Debridement of the necrotic skin and subcutaneous tissue was done. He was transferred to the surgical intensive care unit for further management. The patient was intubated and ventilated. He required a double vasopressor (noradrenaline + vasopressin) to maintain the hemodynamics. Resuscitation was continued with fluids, required blood, and blood products to correct coagulopathy. His renal parameters were deteriorating, and he had metabolic acidosis, hyperkalemia, and serum lactate levels raised to 7. The patient was started on continuous renal replacement therapy (CRRT). He had atrial fibrillation treated with amiodarone infusion for 24 hours.

**Figure 2.**
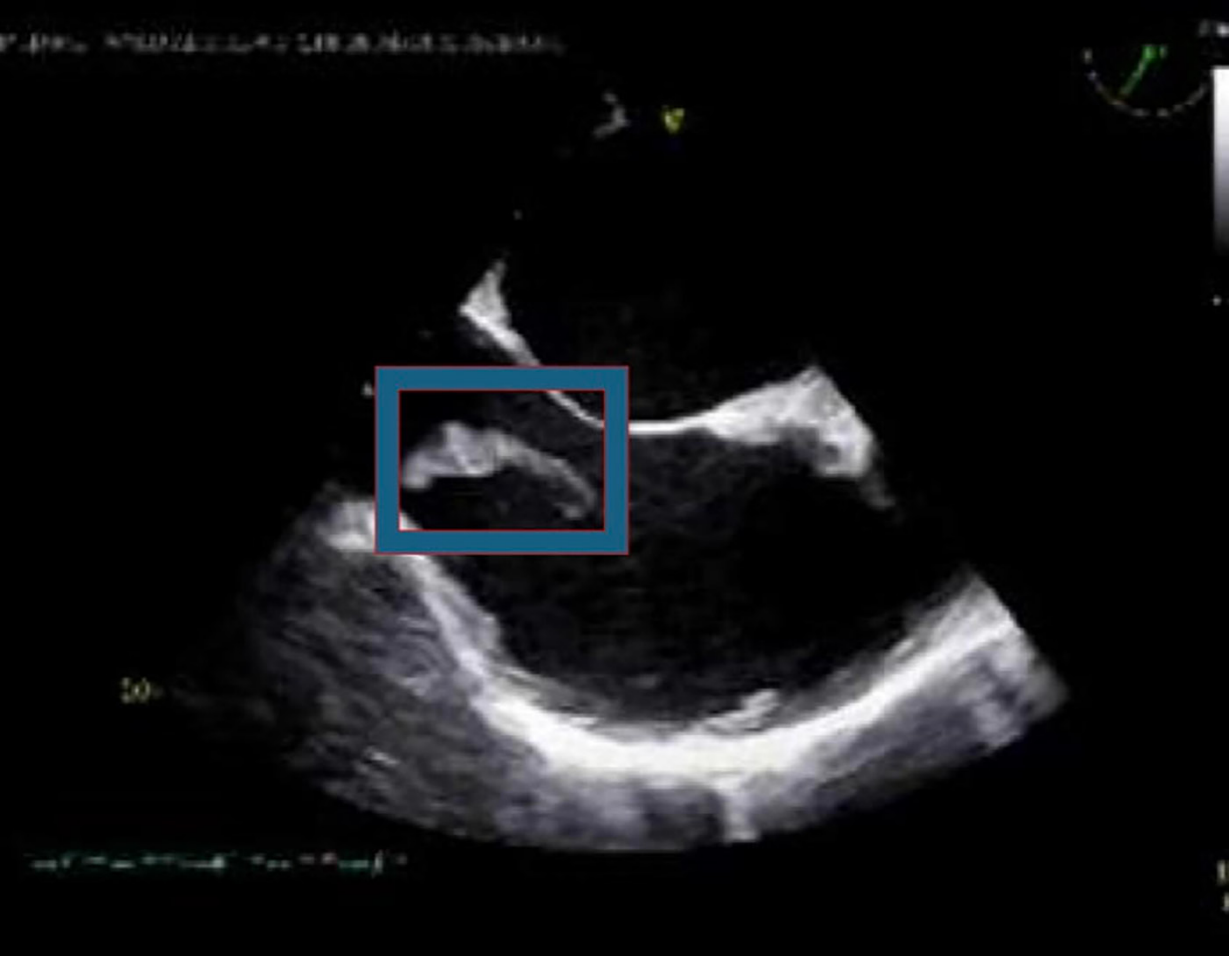
Transesophageal echocardiography showing vegetations.

After 48 hours, the patient was taken for debridement of local necrotic tissue from the sacral and perianal area. The patient remained on both vasopressors, CRRT, and along with continuous resuscitation with fluids, blood, and blood products. He has *E. coli* bacteremia, and the necrotic tissue also grew *E. coli* (ESBL), according to sensitivity to antibiotics, which was changed to meropenem 500 mg once daily intravenously for 6 weeks. On day 3, his renal parameters were improving, he was passing urine, stopped CRRT, and was added furosemide to the therapy. Day 4, he was taken for diversion colostomy to prevent soiling of the wound with fecal matter, and postoperatively started to wean off vasopressors and ventilator. By day 5, the patient improved but had a systolic murmur. Transthoracic echocardiography (TTE; Echo) showed a mobile structure attached to the right atrium with a thread-like attachment in the valve. The transesophageal Echo on day 7 showed right atrium vegetations attached to EV and small filamentous vegetation at the coronary sinus opening. Tricuspid valves were normal (Figure 2). He was fulfilling modified Duke’s criteria for endocarditis.^6^

**Figure 3.**
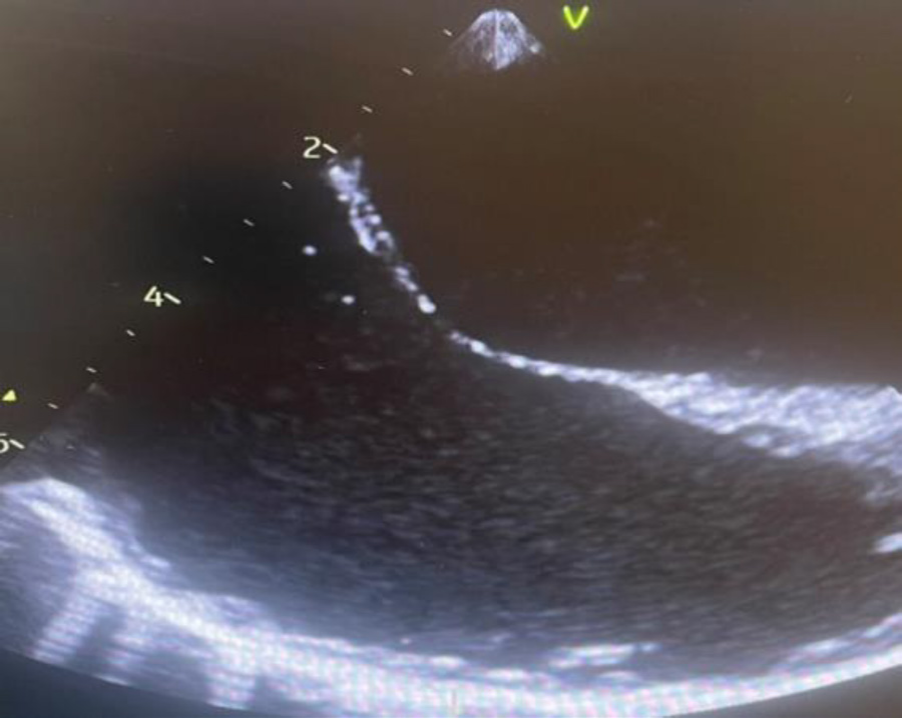
Normal transesophageal echocardiography after discharge.

He continued to be weaned from vasopressors and added heparin infusion for anticoagulation. Microbiology culture showed growth of *Candida dubliniensis*. Anidulafungin (100 mg/day IV) was added to the treatment. His fundoscopic examination of the retina has not shown any abnormality. The patient was built up on enteral feeding, he was successfully liberated from the ventilator and vasopressor by day 9 he was awake. All his invasive remaining lines were removed. Vacuum-assisted closure (VAC) dressing was applied to the wound for further management. He was shifted to the surgical ward on day 12. He continued to receive his antifungal medications and other supportive care. Overall, he was improving from his organ dysfunction. On day 17, he developed pulmonary edema, required furosemide and metoprolol therapy. On day 23, the patient was stable and awake, his wounds were healthy (VAC dressing was removed), tolerated an oral diet. The patient was discharged home on day 27 to receive the antifungal intravenously in the intravenous medication clinic for a total of 6 weeks as an outpatient, along with the surgery clinic and cardiology follow-up. His follow-up echocardiogram (Figure 3) after 2 weeks from discharge showed a normal right atrium and ventricle and complete resolution of the vegetation. The patient is regularly followed in the outpatient clinic for 3 months, and he is doing well.

## 3. DISCUSSION

The large EV is present in approximately 25% of the adult population.^7^ The EV vegetation with right-sided endocarditis is much lower in occurrence compared to the left-sided valve vegetations and endocarditis. The frequent report of vegetation with endocarditis is due to Staphylococcus bacteremia (up to 63% of EV vegetations and endocarditis). It is rare and the only case of EV vegetation due to *Candida* to be reported in the literature; similarly, few cases of *E. coli* (ESBL) of vegetations with endocarditis are reported in the literature.^7^

The detailed search of EVE in PubMed revealed 60 publications, and out of these, 12 publications were either reviews or other types of articles and not case reports or original studies. We were unable to get the details of the four case reports apart from the titles of the case reports. Thus, by analyzing 44 cases of EVE reported in PubMed, we found that implants and indwelling catheters were the major risk factors, followed by intravenous drug abuse and diabetes mellitus, hypertension, and end-stage disease. The microbiological review of these cases showed *Staphylococcus* to be the most common bacterium causing EVE; only three cases of EVE were caused by *Candida*, and two cases were caused by *E. coli*.^9^ Interestingly, only one case of coronary sinus vegetations was reported in all these PubMed cases.^9^

The risk factors for EV vegetations and endocarditis are intravenous drug abuse, implanted cardiac and medical devices or central venous catheters, comorbidities, and immunocompromised individuals, in association with fungemia or bacteremia.^5^

EVE and right-sided endocarditis are rare and underreported. All patients in septic shock with cardiac involvement should have an initial TTE. Around 88% of EV endocarditis is diagnosed with transesophageal echocardiography (TEE). The TEE is more sensitive and specific in the diagnosis of all cases of EVE and right-sided endocarditis.^10^

EVE should be treated with appropriate antimicrobial therapy guided by culture and sensitivity for 6 weeks. The mortality rate in EVE is around 17%. The independent risk factor associated with mortality is acute kidney injury, the Charlson comorbidity index, congestive heart failure, larger vegetation, and central nervous system involvement.^11^

## 4. CONCLUSION

EV vegetation right heart endocarditis with *E. coli* bacteremia and *Candida* fungemia is rare compared to left side endocarditis. Our patient, EVE, was diagnosed quickly, and antimicrobial medications were administered as per culture and sensitivity for 6 weeks. Early diagnosis and longer antimicrobial therapy are essential for better patient outcomes after confirming the diagnosis using appropriate investigations like cultures and transesophageal echocardiogram.
